# The colors of images preferred by individual voxels can be used to delineate functionally distinct visually responsive brain areas

**DOI:** 10.1073/pnas.2535986123

**Published:** 2026-04-07

**Authors:** Ian M. L. Pennock, Chris Racey, Kendrick Kay, Anna Franklin, Jenny M. Bosten

**Affiliations:** ^a^School of Psychology, Sussex Neuroscience, University of Sussex, Falmer BN1 9QH, United Kingdom; ^b^Center for Magnetic Resonance Research, Department of Radiology, University of Minnesota, Minneapolis, Minnesota, MN 55455

**Keywords:** natural scenes, natural scenes dataset, fMRI, functional mapping, color

## Abstract

We exploited co-occurrences between color and other properties of natural scenes to identify and visualize functionally distinct brain regions. For each voxel in the Natural Scenes Dataset (NSD), we computed a scaled response-weighted average of the stimulus images. The colors of “voxel-preferred images” (VPIs) reflect stimulus properties that covary with color in natural scenes: Color serves as a tag for functional distinctions in voxel responses. Mapping VPIs onto cortical surfaces revealed reliable and structured color patterns that segment voxel clusters. Boundaries between clusters of similarly colored VPIs tend to coincide with boundaries defined using other methods, and heterogeneity within regions suggests functional subdivisions. VPIs provide a simple data-driven method for analyzing fMRI responses to natural scenes and visualizing cortical organization.

The visual properties of natural scenes exhibit many statistical “regularities” to which the visual system is thought to be tuned (1). These include relationships among image statistics, and between image statistics and broader image properties such as lighting, objects, and scene categories (2–5). For example, certain objects are linked with particular textures—glass objects are transparent and glossy (6). Colors are salient features that co-occur with many scene attributes, e.g., some object classes have characteristic “canonical” colors, backgrounds tend to be cooler and objects warmer (5), and food objects are associated with warm, saturated colors (4). Interdependencies among scene properties pose a challenge for fMRI researchers seeking to use naturalistic stimuli to investigate brain responses to isolated image properties. However, they also offer opportunities: One property could serve as a marker for neural distinctions linked to correlated scene properties.

We exploited co-occurrences between color and other scene properties in the Natural Scenes Dataset (NSD) (7) to isolate and visualize functionally distinct brain regions. For each voxel, we computed a response-weighted average of the ~10,000 NSD stimulus images seen by the participant (Fig. 1*A*). The resulting “voxel-preferred image” (VPI) reflects low spatial frequency features (e.g., color and luminance) that tend to be present in stimuli that drive the voxel. Mapping VPIs onto the cortical surface revealed structured patterns, in which clusters of similarly colored voxels distinguished regions with common preferences for image properties correlated with color in the NSD stimuli. These color patterns were reliable within and between participants and delineate functionally distinct cortical regions.

**Fig. 1.**
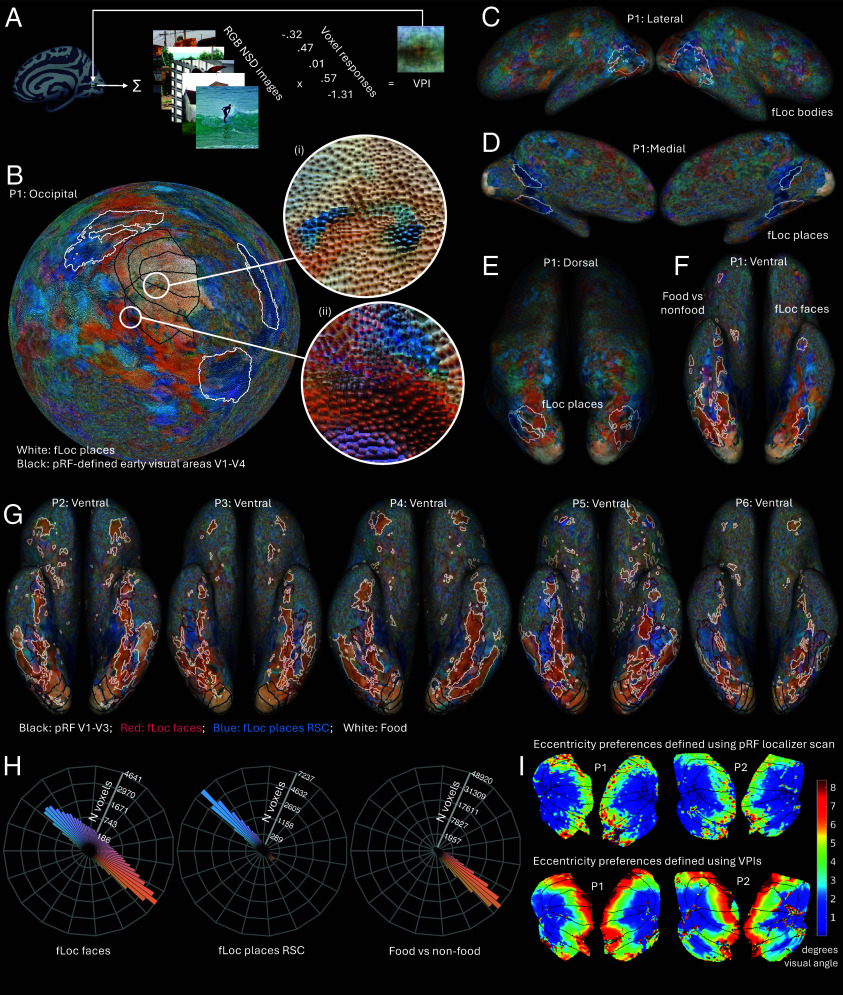
VPIs across the cortical surface. (*A*) VPI method: For each voxel, stimulus images are summed, weighted by the voxel’s response to each image. The resulting weighted-sum color image matrix (VPI), rescaled to 0 to 255 for visualization, is plotted at the voxel’s cortical location. (*B*) Spherical occipital view showing VPIs for Participant 1 (P1), with pRF-mapped early visual areas (black contours), and functionally mapped place-selective areas (white contours). Zoomed regions: i) ventral V1/V2 near the fovea; ii) V4-LO2/PIT boundary. (*C*–*F*) Cortical views for P1 plotting VPI median colors with ROI contours: (*C*) lateral; (*D*) medial; (*E*) dorsal; (*F*) ventral. Panel (*F*) shows hemisphere-specific ROI contours. (*G*) Ventral views for five participants with labeled ROI contours. (*H*) Distributions of median VPI colors for labeled ROIs across all participants (radial distance ∝ √N voxels). (*I*) Comparison of V1–V4 eccentricity preferences derived from VPIs and the pRF localizer.

## Results

**Fig. 2.**
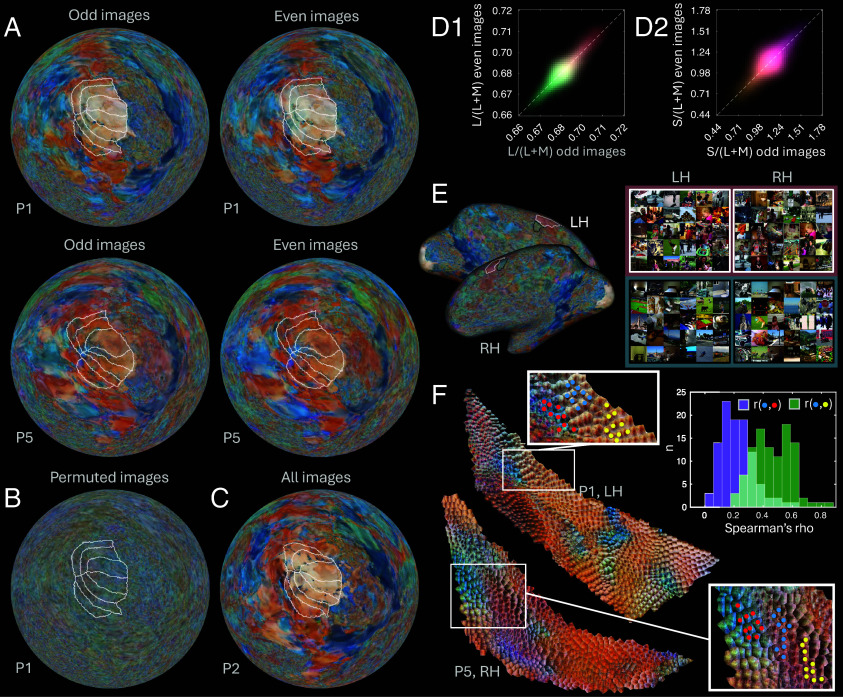
Reliability and functional significance of VPI color patterns. (*A*–*C*) fsaverage cortical maps of median VPI colors with pRF-mapped V1–V4: (*A*) based on independent split halves of images (for P1 and P5); (*B*) with permuted image-beta correspondences; (*C*) for P2. (*D*) Split-half reliabilities for L/(L+M) (d1) and S/(L+M) (d2) median VPI chromaticities across all participants. For each 2D histogram, luminance reflects the cube root of frequency. (*E*) Pink and adjacent bluish ROIs for P1 with corresponding montages of the 30 stimuli eliciting the greatest average response in each ROI. (*F*) For V4 in both hemispheres and all participants we defined triplets of voxel clusters where Clusters A (yellow dots) and B (blue dots) share a VPI color, and Cluster C (red dots) differs from *B*. The histogram shows RDM correlations for clusters with shared (green bars) versus distinct (purple bars) VPI colors.

We created VPIs for all voxels, which are plotted for a participant inside Voronoi cells on a spherical cortical surface centered on the occipital pole (Fig. 1*B*). Across the cortex, VPI colors form striking structured patterns through ventral and dorsal visual pathways and into frontal regions (Fig. 1 *C*–*G*), which are not visible in simpler maps of maximally activating stimuli. Most VPIs show a colored “blob” on a differently colored background (Fig. 1*B*). In V1–V4, dark blobs on pale backgrounds may reflect OFF-pathway dominance (8). Blob positions covary with voxel pRFs [circle (i) of Fig. 1*B*]: VPI-derived eccentricity and angle estimates correlated with independent NSD pRF measures (mean ρ_ecc_ = 0.69 for LH, 0.70 for RH; mean ρ_ang_ = 0.50 for LH, 0.53 for RH; all *P* ≪ 0.0001 Fig. 1*I*). Beyond V1–V4, VPI colors show systematic heterogeneity, with frequent blues and oranges [likely reflecting their dominant variance in natural scenes (9)], and clusters of purples, reds, and greens (Figs. 1 *B*–*G* and 2 *A* and *C*).

Patterns of VPI colors are highly reliable when created using independent split halves of the NSD images (Fig. 2*A*). Across participants, there were strong commonalities alongside some individual differences (Figs. 1*G* and 2 *A* and *C*). To quantify reliability, we converted VPI median RGBs to MacLeod–Boynton chromaticity coordinates (Fig. 2*D*). Mean whole-brain split-half correlations were *r* = 0.78 (range: 0.70 to 0.87; Fig. 2 *D, i*) for L/(L+M), and *r* = 0.65 (range: 0.46 to 0.80; Fig. 2 *D, ii*) for S/(L+M), indicating high reliability. As a control for spatially correlated noise, we created VPIs with permuted image-beta parings, which showed no coherent or reliable color patterns (Fig. 2*B*).

Spatial clusters of similar VPI colors reflect shared image properties correlated with color in the NSD stimuli, provoking similar voxel responses. Boundaries of functionally defined regions localized by other methods tend to coincide with these VPI clusters: pRF-defined regions (7) align with whitish VPIs (Fig. 1*B*), food-selective regions (4) with orangish VPIs (Fig. 1 *F*–*H*), and body- and place-selective regions (7), though less clearly, with light blue (Fig. 1*C*) and dark blue (Fig. 1 *D* and *F*–*H* VPIs. Within areas such as V4 (Fig. 1*B*) and face-selective areas (Fig. 1 *F*–*H*), heterogenous VPI colors form spatial subclusters that may indicate functional subdivisions.

To illustrate how the functions of VPI-defined ROIs can be explored, we compared neighboring ROIs defined by distinct VPI colors and visualized montages of the stimuli eliciting the strongest average voxel responses. For the example pink ROIs in Fig. 2*E*, 70 of the top 100 images for LH (*P* < 0.0001 based on a permutation test) and 81 for RH (*P* < 0.0001) contained babies or children. For the adjacent bluish ROIs none of the top 100 images for LH (*P* = 0.0048) and only three for RH (*P* = 0.25, n/s) contained babies or children.

We investigated the functional significance of small voxel clusters with a common VPI color in V4. For each participant and hemisphere, we manually identified triplets of 10-voxel contiguous clusters (A, B, and C) using VPIs based on odd-numbered stimulus images: Clusters A and B shared a VPI color but were separated by at least 1 voxel; C differed in color and was similarly separated from B (Fig. 2*F*). For each cluster, we computed an RDM based on voxel responses to even-numbered stimulus images, and correlated A–B and B–C RDMs. Over 101 triplets, correlations were significantly greater for A–B than for B–C RDMs [*t*(100) = 14.9, *P* = 3.3 × 10^−27^; Fig. 2*F*]. Differences in RDM similarity were not due to cortical distance: Geodesic distances between A–C and B–C cluster centers did not significantly differ [*t*(100) = 0.41, *P* = 0.68; mean A–B = 3.18 mm; mean B–C = 3.13 mm]. Thus, distinct VPI colors are a marker of functional distinctions between voxel clusters.

## Discussion

By using color to tag responses to correlated image properties, we found reliable structured patterns of functional organization across the cortex, visualized as systematic variations in VPI color. Individual VPIs offer visualization of pRFs similarly to conventional grayscale pRF models, but the inclusion of color reveals chromatic pRFs, often appearing as a colored blob on a contrasting background. For most voxels, VPI colors reflect preferences for scene properties correlated with those colors, e.g., the pink ROI in Fig. 2*E* may relate to correlations between children and pink clothing or environments. For some voxels, true color-sensitive responses might also contribute to VPI colors: Such contributions would need dissociating from responses driven by correlated image properties using controlled stimuli. Interestingly, VPI colors in color-responsive V4 show heterogeneity aligned with functional subdivisions, consistent with recent evidence of topographic scene representations in macaque V4 (10).

A key difference between the VPI method and other data-driven approaches such as PCA or nonnegative matrix factorization (11, 12) is that VPIs are explicitly stimulus-linked, improving interpretability, and separating stimulus-related responses from other structured voxel activity. VPIs could also be applied to datasets using stimuli with controlled contingencies between color and other stimulus features, supported by recent evidence that color helps functionally segment voxels in macaque early visual areas (13). In addition to tagging functional distinctions, VPI colors exploit the power of human visualization for parcellating cortical maps. By exploiting contingencies between color and other image properties in natural scenes, VPIs offer a new intuitive and scalable method for exploring large fMRI datasets to reveal functional structure that is otherwise difficult to detect.

## Materials and Methods

We analyzed the open-access 7T fMRI NSD (7), which provides fMRI data from eight participants who viewed ~10,000 natural scene images across 30 to 40 sessions. Our analyses also incorporated data from NSD functional localizer and pRF mapping scans. Please see the *SI* *Appendix* for further details.

## Supplementary Material

Appendix 01 (PDF)

## Data Availability

The NSD is at https://naturalscenesdataset.org (14). Scripts to reproduce all results, with versions of Figs. 1 *C*–*G* and *I* and 2*A* for all participants are at https://osf.io/v5wxn/ (15).
