# Supplementary material for: The colors of images preferred by individual voxels can be used to delineate functionally distinct visually responsive brain areas
Source: Proc Natl Acad Sci U S A. 2026 Apr 7;123(15):e2535986123. doi: 10.1073/pnas.2535986123 (PMC13080022; doi:10.1073/pnas.2535986123)
Supplement: Supplementary file 1 — Appendix 01 (PDF) [file pnas.2535986123.sapp.pdf]

Supporting Information

Extended Methods

We exploited the co-occurrences of colors with other properties of natural scenes to use color to 'tag' functional distinctions between brain areas in fMRI voxel responses to natural scene stimuli. To do this we created a 'voxel-preferred image' (VPI) for each voxel, a scaled weighted average of the natural scene stimulus images, weighted by voxel responses (see 'Generation of voxel-preferred images'). The VPI is an RGB image that reflects the color commonalities in the natural scene images that tend to provoke positive responses in the voxel (and opposite to color commonalities that tend to provoke negative responses). When VPIs or the average colors of VPIs are plotted at the corresponding voxel locations on the cortical surface, color boundaries reflect changes in functional responses to a property or properties of the natural scene stimuli correlated with stimulus color in the stimulus set.

Natural Scenes Dataset

We used the Natural Scenes Dataset, a large open-access fMRI dataset. For detailed accounts of data acquisition and preprocessing, see Allen et al. (2022).

Participants

Eight participants (six females; age range 19-32 years) were included in the Natural Scenes Dataset; all had normal or corrected-to-normal vision. The University of Minnesota Institutional Review Board approved the experimental protocol and informed consent was obtained. All human data used in the present study were de-identified and publicly available – additional ethical approval was therefore not required.

Stimulus display

Stimuli were presented on a BOLDscreen 32 LCD monitor (Cambridge Research Systems, Rochester, UK) with a spatial resolution of 1920 by 1080 pixels. The participants' eyes were 5 cm from a mirror which was 171.5 cm from the monitor. A PR-655 spectroradiometer (PhotoResearch, Chatsworth, CA) was used to measure the spectral power distributions of the display primaries, to color calibrate the BOLDscreen and allow a calibrated transformation from RGB to LMS tristimulus cone activities.

Stimuli

The natural scene images were a subset of RGB images in the Microsoft Common Objects in Context (COCO) dataset (2). The images contained 80 categories of object (e.g., faces, cars, foods, stop signs, animals). The NSD images were square crops of 425 x 425 pixels extracted from the original COCO images. They were up-sampled for presentation on the BOLDscreen to 714 x 714 pixels where they subtended 8.4 x 8.4 degrees of visual angle. The BOLDscreen provides a linear relationship between light intensity and RGB values. To provide a gamma similar to that imposed by most displays, NSD image RGB values were squared before presentation.

For each participant there were 10,000 distinct natural scene images intended to be shown 3 times each over 40 scan sessions. Participants 1, 2, 5 and 7 completed all 40 planned scan sessions. Participants 3 and 6 completed 32 scan sessions and saw 9,411 distinct images, and participants 4 and 8 completed 30 scan sessions and saw 9,209 distinct images. Each distinct image was intended to be shown 3 times, but for participants who did not complete all 40 scan sessions some images were shown fewer than 3 times. Of the 10,000 distinct images assigned to each participant, 1000 were common across all 8 participants and 9,000 were unique for that participant.

Localizer experiments

In an additional scan session there were additional experiments to estimate population receptive fields (pRFs) in retinotopic areas and to map functional regions (fLoc). In the pRF experiment, dynamic

colored textures were presented in moving apertures which varied in x or y directions or along polar axes. In the fLoc experiment, greyscale images of five categories of visual stimulus were presented (3): characters (words and numbers), bodies, faces, places (houses and corridors) and objects (cars and musical instruments). Objects were presented on scrambled backgrounds and stimuli had the same dimensions as the natural scene stimuli. Resulting pRF parameter estimates (fit using a standard model), estimates of retinotopic visual areas V1-V4, and estimates of functional regions for each fLoc stimulus class are available as part of the NSD dataset.

Natural scene stimulus experiment
During the 'main task' in the scanner participants were shown the natural scene stimuli, and had to judge whether each image was new or had been presented previously. On each trial an image was shown for 3 s with a semi-transparent red fixation dot on a grey background. Then, for 1 s, the red fixation dot was shown on the grey background only.

Scanner
Participants were scanned using a 7T Siemens Magnetom scanner at the University of Minnesota with a single channel transmit 32-channel receive RF head coil. A gradient-echo EPI sequence was used at 1.8 mm isotropic resolution (whole brain; 84 axial slices, slice thickness 1.8mm, slice gap 0 mm, field-of-view 216 mm (FE) × 216 mm (PE), phase-encode direction anterior-to-posterior, matrix size 120 × 120, TR 1600 ms, TE 22.0 ms, flip angle 62°, echo spacing 0.66 ms, bandwidth 1736 Hz/pixel, partial Fourier 7/8, in-plane acceleration factor 2, and multiband slice acceleration factor 3).

Pre-processing
Full pre-processing procedures are described in Allen et al. (1). Data were temporally resampled to correct for slice time acquisition differences. The resampled volumes were undistorted using field estimates from field maps. SPM5 spm_realign was then used with the volumes to estimate rigid-body motion parameters. Head motion and spatial distortion were corrected using a single cubic interpolation on the resampled volumes. The mean fMRI volume was calculated and corrected for gradient nonlinearities. This was then co-registered to the gradient-corrected volume from the first scan session, to provide the target space for preparing fMRI data from the different scan sessions.

A GLM analysis was applied to the fMRI time-series data to estimate single-trial beta responses. We used the b3 version of the NSD betas ('betas_fithrf_GLMdenoise_RR'; native surface space), which were pre-processed as described in Allen et al. (1). In brief, the GLMsingle algorithm (1, 4–6) was used to derive nuisance regressors and to choose the optimum ridge regularization shrinkage fraction for each voxel. The betas represent estimates of the trial-wise BOLD response amplitudes to each stimulus trial for each voxel, relative to the average BOLD signal observed during blank trials (when only the grey screen was shown). Betas for trials presenting the same image were averaged to improve signal estimates and reduce the size of the dataset – we refer to these as 'average image trials'. All analyses were done in MATLAB (MathWorks Inc., Natick, USA).

Generation of voxel-preferred images
To compute voxel-preferred images (VPIs), we first z-scored the voxel responses for each individual voxel within each scan session. To reduce computational load, the NSD images were downsampled by retaining every 6th pixel, making the original 425 x 425 pixel images 71 x 71. Equation 1 describes the creation of VPIs. For each voxel, the RGB values of each image (I) were weighted by the corresponding z-scored voxel response to that image (β), and then summed over all available images per participant (N_I).

$$\text{VPI} = \sum_{I=1}^{I=N_I} \beta I \qquad \text{Equation 1}$$

The final VPI was then rescaled so that its minimum was 0 and its maximum 255 for visualization in RGB. We calculated the median colors of VPIs as the median from each RGB channel over the whole VPI.

VPI surface plots

To plot thumbnails of voxels' VPIs at their cortical locations (e.g., Figure 1b), we computed Delaunay triangles for cortical surface voxel coordinates and then the Voronoi diagram for the vertices of the Delaunay triangles. We scaled each VPI to be the same size as the maximum excursion in x and y of the associated Voronoi cell and then used the Voronoi cell for the voxel as a mask for the VPI. We then plotted each masked VPI at the cortical surface location of its voxel. For cortical surface maps of median VPI colors (e.g., Figure 1c) we calculated the median RGB value of each voxel's 71 x 71 x 3 VPI. We then plotted 3 surface maps for the values of the R, G and B median VPI colors at the locations of each corresponding voxel, then combined the three image layers into one RGB cortical surface map.

ROIs for retinotopically defined areas, fLoc-defined areas and food selective areas

The pRF-defined contours plotted in Figure 1b and the face, place and body ROI contours plotted in Figure 1c are provided as part of the NSD dataset (1), and result from the additional pRF and fLoc experiments summarized above.

For the food-selective regions plotted in Figure 1c, we used the *T*-values for the difference in voxel responses between images containing food and images containing no food, and then ran a multiple linear regression on each positive significant voxel from that analysis (7). We defined the food-responsive ROIs as containing each significant voxel where food had the largest positive beta coefficient out of a set of image statistics that were included in the multiple linear regression (see (7) for the other image statistics). To ensure that our food-selective ROIs were defined independently of the VPIs themselves, we used even average image trials to calculate the VPI colors plotted in Figures 1f and 1g, and used odd average image trials to define the food-selective ROI contours.

Polar hue histograms

Polar hue histograms plotted in Figure 1h show the distributions of median VPI colors for three functionally defined ROIs. Median VPI colors for voxels within the ROIs, defined in participant native surface space, were concatenated for all 8 NSD participants and both hemispheres. $\sqrt{N}$ voxels is plotted radially, to allow better visualization of rarer VPI colors. Each histogram wedge is colored according to the bin's mean hue and according to the mean saturation of median VPI colors falling into that hue bin. To ensure that food-selective ROIs were defined independently of the median VPI colors, we plotted VPI colors defined using even average image trials, and used odd average image trials to define the ROIs.

Extraction of pRF parameters from VPIs

To estimate pRF parameters from VPIs, we first inverted each VPI in areas V1-V4 (for which the NSD has localizer data from the independent pRF mapping experiment), and scaled it from 0-1. To each inverted scaled VPI we fit a bivariate Gaussian, and then extracted the coordinates of the center of the fitted bivariate Gaussian. We converted these coordinates to polar coordinates (with respect to the VPI center) to provide estimates of pRF angle and eccentricity. We then mapped the derived angle and eccentricity preferences to the cortical surface and compared them with similar preference maps derived from the NSD's pRF mapping experiment (e.g., Figure 1i). We calculated the Spearman correlation between eccentricity preferences derived from the two methods for all participants and both hemispheres, and found the average Fisher-transformed correlation coefficients. The mean correlations reported are the inverse Fisher-transformed averages.

Permuted VPIs

For permuted VPIs, we randomly permuted the order of the images with respect to the betas to break the link between betas and images. We then calculated VPIs in the same way as for the original (unpermuted) data.

Reliability analyses

For the split-half reliability analyses, we split the average image trials into two groups: odd and even. Each group had 4,604 to 5,000 unique natural scene images in them (depending on the number of distinct images each participant saw). We calculated VPIs and median RGBs for VPIs separately for the odd and even image groups. To quantify the split-half reliability of the median colors of VPI images, we then converted the median VPI RGB values to MacLeod-Boynton (8) chromaticity coordinates S/(L+M) and L/(L+M) using a transform matrix based on the measured display RGB spectra. We plotted values derived from odd images against values derived from even images for each voxel and conducted Pearson correlations for both S/(L+M) and L/(L+M).

Stimulus montages

To plot the montages shown in Figure 2e, we calculated mean betas for each stimulus image over all the voxels in each ROI. For each ROI, we then identified the first N ranked stimulus images for mean beta. We then plotted those stimulus images in montages ranging from the first ranked stimulus image to the last ranked stimulus image over the set (along columns then rows from left to right). N was 100 for the results reported in the main text and 30 for the figure, to allow for visible image thumbnails in the montages.

Permutation analysis

For the permutation tests reported for the ROIs shown in Figure 2e, we conducted 10,000 permutations of an analysis to count the number of babies and children in the two ROIs (marked white and black in Figure 2e), separately for the left and right hemispheres. To count the number of babies and children in the ROIs for the original (unpermuted) data, we used the manually defined ROIs based on median VPI color as masks to select the set of ROI betas for average image trials. We calculated the mean beta per average image trial for each ROI, then (as for the stimulus montages), extracted the 100 highest mean betas for each ROI. We had separately manually labelled each NSD stimulus image as containing babies, children or adults (or none). We summed the number of the 100 most activating images that contained babies or children for each ROI.

For the permutation analysis, we repeated the procedure over 10,000 permutations, using a set of betas for each ROI where the order of image average trials had been randomly permuted. Over the permutations, we summed the number of the 100 'most activating images' that contained babies or children for each ROI. We used the distribution of permuted sums to estimate the probability that the observed number of images containing babies or children arose by chance.

hV4 analysis

To investigate the functional significance of boundaries between cortical areas with different VPI colors, we analyzed representational dissimilarity for sub-regions of left and right hemisphere hV4 defined by VPI color. We first defined LH and RH hV4 for all 8 NSD participants from results of the NSD pRF localizer experiment (see 'Localizer experiments'). For each participant we plotted cortical surface maps of VPIs in left and right hemisphere hV4 masked by voxel Voronoi cells at voxels' cortical surface locations (e.g., Figure 2f). From these maps we then selected 101 sets of 30 voxels. Within each set of 30 voxels, Group A of 10 voxels had VPIs of an approximately consistent VPI color and formed one contiguous area on the cortical surface. Group B of 10 voxels had approximately the same VPI color as Group A. Group B also formed a contiguous area on the cortical surface and was separated from Group A by 1 voxel at its closest point. Group C of 10 voxels had a consistent VPI color within the group, but had a *different* VPI color from voxels in Group B. Group C also had a contiguous area on the

cortical surface and was separated from Group B set by 1 voxel at its closest point. Thus, among the set of 30 voxels were 3 groups of 10 voxels, which were divided into two comparison groups. Groups A vs B and Groups B vs C. The two comparison groups were matched on cortical surface distance (between the two component groups of 10 voxels), but Groups A and B had similar VPI colors while Groups B and C had different VPI colors. Voxels were manually selected based on judged similarity of the colors of VPIs plotted within Voronoi cells. Voxels were selected before running any analysis of the RDMs (i.e., RDMs were not known or used for voxel selection). To ensure that ROI definition was based on independent data from the RDMs, we selected ROIs based on VPIs created using odd numbered average image trials, and created RDMs for even numbered average image trials.

We estimated the spatial separations between A vs B and B vs C voxel groups by computing geodesic distances along the convoluted cortical surface (using the FreeSurfer white matter surface mesh). For each manually drawn ROI, a centre of mass was identified by computing a geodesic distance matrix of all possible coordinate pairs for the vertices within the region. The vertex with the minimum sum of distances to all other vertices was selected as the ROI centroid. The shortest paths between A vs B ROI centroids and between the B vs C ROI centroids were calculated using MATLAB's graph-based search. The resulting cortical distance measurements are reported in mm and respect the topology of the cortical surface. To statistically compare A vs B and B vs C cortical distances, we conducted a matched-pairs T-test on the 101 pairs of distances derived from the 101 ROI triplets.

Betas from each group of 10 voxels were then used to calculate Euclidian distance RDMs for the even numbered images shown to the corresponding participant. For each of the 101 sets of voxels, three RDMs were created, one for each group of 10 voxels within the set. Comparisons were then made by calculating Spearman correlations between the RDM for Group A voxels and the RDM for Group B voxels, and between the RDM for Group B voxels and the RDM for Group C voxels. Finally, Spearman correlations for Groups A vs B RDMs were then compared with Spearman correlations for Groups B vs C RDMs. If VPI color is a guide to functional distinctions, Groups A vs B Spearman correlations should be larger than Groups B vs C Spearman correlations.

References

1. E. J. Allen, *et al.*, A massive 7T fMRI dataset to bridge cognitive neuroscience and artificial intelligence. *Nat Neurosci* (2022). https://doi.org/10.1038/s41593-021-00962-x.

2. T. Y. Lin, *et al.*, *Microsoft COCO: Common objects in context* (2014).

3. A. Stigliani, K. S. Weiner, K. Grill-Spector, Temporal Processing Capacity in High-Level Visual Cortex Is Domain Specific. *J. Neurosci.* **35**, 12412–12424 (2015).

4. K. N. Kay, A. Rokem, J. Winawer, R. F. Dougherty, B. A. Wandell, GLMdenoise: A fast, automated technique for denoising task-based fMRI data. *Front. Neurosci.* **7**, 1–15 (2013).

5. A. Rokem, K. Kay, Fractional ridge regression: a fast, interpretable reparameterization of ridge regression. *arXiv* 1–12 (2020). https://doi.org/10.1093/gigascience/giaa133.

6. J. S. Prince, *et al.*, GLMsingle: a toolbox for improving single-trial fMRI response estimates. *bioRxiv* **5000**, 2022.01.31.478431 (2022).

7. I. M. L. Pennock, *et al.*, Color-biased regions in the ventral visual pathway are food selective. *Curr. Biol.* **33**, 134-146.e4 (2023).

8. D. I. MacLeod, R. M. Boynton, Chromaticity diagram showing cone excitation by stimuli of equal luminance. *J. Opt. Soc. Am.* **69**, 1183–6 (1979).
